# Explantation and Guided Repositioning of Two Mandibular Implants for Rehabilitation With an Implant-Mucosa-Supported Overdenture in a Patient With Advanced Peri-Implantitis: A Case Report

**DOI:** 10.7759/cureus.112422

**Published:** 2026-07-10

**Authors:** Karina del Rocío Carreri Santiago, Pedro Alberto López Reynoso, Hebert Basulto Diaz, Cesar Esquivel Chirino, Daniela Carmona Ruiz

**Affiliations:** 1 Advanced Restorative Dentistry, División de Estudios de Posgrado e Investigación, Facultad de Odontología, Universidad Nacional Autónoma de México, Mexico City, MEX; 2 Basic Medical Sciences, Facultad de Odontología, Universidad Nacional Autónoma de México, Mexico City, MEX; 3 Orthodontics, Facultad de Odontología, Universidad Nacional Autónoma de México, Mexico City, MEX

**Keywords:** dental implants, implant-supported overdenture, maintenance of peri-implant health, periimplantar diseases, periimplantitis, surgical explantation

## Abstract

Peri-implantitis is a biological complication that can compromise long-term implant survival and, in advanced cases, may indicate explantation. In patients with mandibular edentulism rehabilitated with implant-retained overdentures, the management of advanced peri-implant disease is particularly challenging when systemic factors and unfavorable peri-implant tissue conditions coexist. We report the case of a 73-year-old woman with a medical history of rheumatoid arthritis and Sjögren’s syndrome who was wearing an implant-retained mandibular overdenture supported by two intraforaminal implants. She presented with prosthetic instability, peri-implant pain, and clinical and radiographic findings consistent with a Class IIIc combined peri-implant bone defect with advanced severity at the left mandibular site. Treatment included explantation of the affected implants followed by digitally planned, prosthetically guided immediate placement of two new intraforaminal implants. A subsequent soft tissue augmentation procedure with de-epithelialized free gingival grafts was performed to increase the band of peri-implant keratinized mucosa. Definitive rehabilitation was completed with an implant-mucosa-supported overdenture retained by low-profile Equator attachments (Rhein83, Bologna, Italy). At three months after loading, clinical and radiographic evaluation showed peri-implant stability, healthy soft tissues, and adequate prosthetic retention and function. The short follow-up does not allow conclusions regarding long-term outcomes, but the case illustrates a feasible workflow for managing advanced peri-implantitis in a medically compromised patient when conservative or regenerative strategies are considered unpredictable.

## Introduction

Edentulism remains a public health condition with substantial impact on quality of life, since it limits essential functions such as feeding, phonation, and facial appearance [1]. Implant-supported rehabilitation is an effective alternative for the management of edentulous patients because it improves prosthetic stability and helps preserve residual bone volume compared with conventional removable prostheses [2]. The long-term success of dental implants, however, depends not only on adequate osseointegration but also on biological factors related to aging that may influence wound healing and the peri-implant bone response [3].

After tooth loss, the alveolar ridge undergoes a remodeling process characterized by progressive reduction of horizontal and vertical dimensions, particularly during the first months after extraction [4]. Contemporary implant therapy has shifted from a perspective focused exclusively on surgical placement to maintenance-oriented strategies aimed at preserving peri-implant tissue health [5]. Among the most frequent biological complications associated with dental implants are peri-implant mucositis and peri-implantitis. According to the 2017 World Workshop, peri-implant mucositis is characterized by inflammation of the soft tissues without bone loss, while peri-implantitis involves inflammation associated with progressive bone loss [6]. Recent studies have reported approximate prevalence rates of 43% for peri-implant mucositis and 22% for peri-implantitis [7]. Bacterial biofilm accumulation is widely recognized as the main etiological factor in the development of these conditions, particularly in the absence of an adequate peri-implant maintenance program [8].

Several prosthetic options are available for rehabilitating the edentulous mandible, including the conventional complete denture, the implant-retained overdenture supported by two or more implants, the bar-retained multi-implant overdenture, and the fixed full-arch implant-supported prosthesis. The choice among them depends on residual bone volume, functional demands, manual dexterity, hygiene capacity, systemic status, and cost. Among these, the two-implant overdenture is regarded as the minimum standard of care for the edentulous mandible, offering a favorable balance between improved retention and stability, surgical simplicity, ease of maintenance, and affordability [9].

When the retention of a conventional complete denture is deficient, the placement of two intraforaminal implants to support an overdenture has been shown to improve prosthetic stability, masticatory function, and patient satisfaction, and is currently considered the minimum standard of care for the edentulous mandible. According to the McGill Consensus, edentulous mandibular patients should not be rehabilitated exclusively with conventional dentures, and at least two implants supporting an overdenture are recommended [9]. Implant overdentures, however, have specific maintenance challenges, since retention systems may favor biofilm and calculus accumulation when adequate hygiene protocols and periodic professional follow-up are not implemented [10,11].

Long-term preservation of peri-implant health requires an integrated preventive protocol including periodic clinical and radiographic evaluation of the peri-implant tissues, control of local and systemic risk factors, professional removal of biofilm and retentive factors, patient education on mechanical biofilm control, and timely treatment of peri-implant disease [5]. When these strategies fail and peri-implantitis progresses, the affected implants may become compromised and, in advanced cases, surgical interventions such as explantation may be required. This scenario is particularly demanding in older adults with systemic conditions that may affect wound healing and biofilm control.

The aim of this case report is to describe the clinical management of advanced peri-implantitis through explantation of the affected implants and prosthetically guided placement of new intraforaminal implants. Rehabilitation was performed with an implant-mucosa-supported overdenture retained by Equator axial attachments (Rhein83, Bologna, Italy), with the goal of improving stability and function. The case also emphasizes patient education on oral hygiene and structured peri-implant maintenance to reduce the risk of recurrence. Digital, prosthetically guided planning has been associated with more accurate three-dimensional implant positioning and may improve the predictability of functional and esthetic outcomes, which supported its use in the present case.

## Case presentation

A 73-year-old woman attended the Advanced Restorative Dentistry Specialty Program at the Faculty of Dentistry in the Postgraduate Studies and Research Division, Faculty of Dentistry, National Autonomous University of Mexico, reporting pain in the anterior mandibular region, difficulty inserting her mandibular overdenture, and progressive deterioration of masticatory function. The medical history included rheumatoid arthritis and primary Sjögren’s syndrome, both pharmacologically treated and clinically controlled at the time of evaluation. Primary Sjögren’s syndrome is associated with salivary gland dysfunction and reduced salivary flow, which lowers mechanical biofilm clearance and the antimicrobial and buffering capacity of saliva and may favor peri-implant biofilm accumulation; rheumatoid arthritis and its immunomodulatory treatment may further influence the inflammatory response and soft-tissue healing. These conditions were therefore considered relevant modifiers of peri-implant disease risk and of surgical and prosthetic planning. The patient had been wearing a mandibular overdenture retained by two intraforaminal implants placed several years before, with no documented record of structured peri-implant maintenance after delivery. Written informed consent for the diagnostic procedures, the proposed treatment, and the publication of clinical information and photographs was obtained from the patient.

**Figure 1:**
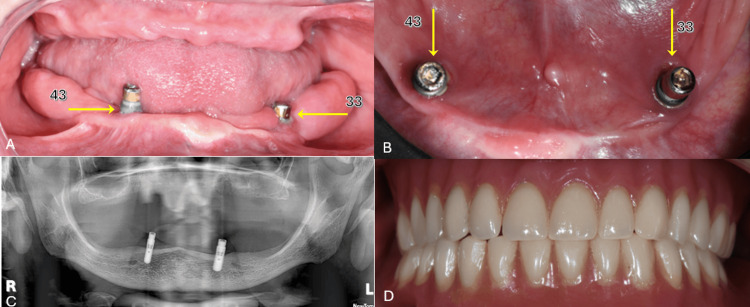
Initial diagnostic assessment. (A-B) Peri-implant intraoral photographs showing soft tissue inflammation and absence of keratinized mucosa (yellow arrows). (C) Diagnostic orthopantomography showing bilateral peri-implant bone loss. (D) Patient’s maxillary and mandibular complete dentures.

Initial intraoral peri-implant photographs (Figures 1A, 1B) showed peri-implant inflammation, loss of soft tissue architecture, and the absence of an adequate band of peri-implant keratinized mucosa around both implants. The diagnostic panoramic radiograph (Figure 1C) revealed bilateral peri-implant bone loss; the maxilla was edentulous and rehabilitated with a conventional complete denture, which served as the antagonist arch (Figure 1D).

All peri-implant probing depths and keratinized mucosa widths were recorded by a single calibrated examiner using a UNC-15 periodontal probe; radiographic peri-implant bone levels were measured on Cone-beam computed tomography (CBCT) with Mimics software (Materialise, Leuven, Belgium) and Blue Sky Plan (Blue Sky Bio, Libertyville, IL, USA) following a standardized protocol. Circumferential peri-implant probing depths were 7 mm at implant 33 and 5 mm at implant 43, with bleeding on probing and exposure of the implant threads. The width of peri-implant keratinized mucosa at baseline was approximately 1 mm at the buccal aspect of implant 33 and 3 mm at the buccal aspect of implant 43. Based on the clinical and three-dimensional radiographic findings, the bone defects were classified as combined peri-implant defects (Class III) with a circumferential component (subclass c) following the morphological and severity classification of peri-implantitis bone defects proposed by Monje et al. [12]; the severity grade was advanced (greater than 50% of vertical bone loss relative to implant length) at implant 33 and moderate (between 25% and 50%) at implant 43. Vertical bone loss measured on CBCT was approximately 7 mm at implant 33 and 6 mm at implant 43. The mandibular ridge was classified as Atwood Class IV [13], and bone quality at the recipient site was estimated as type D3 according to the Misch classification [14].

**Figure 2:**
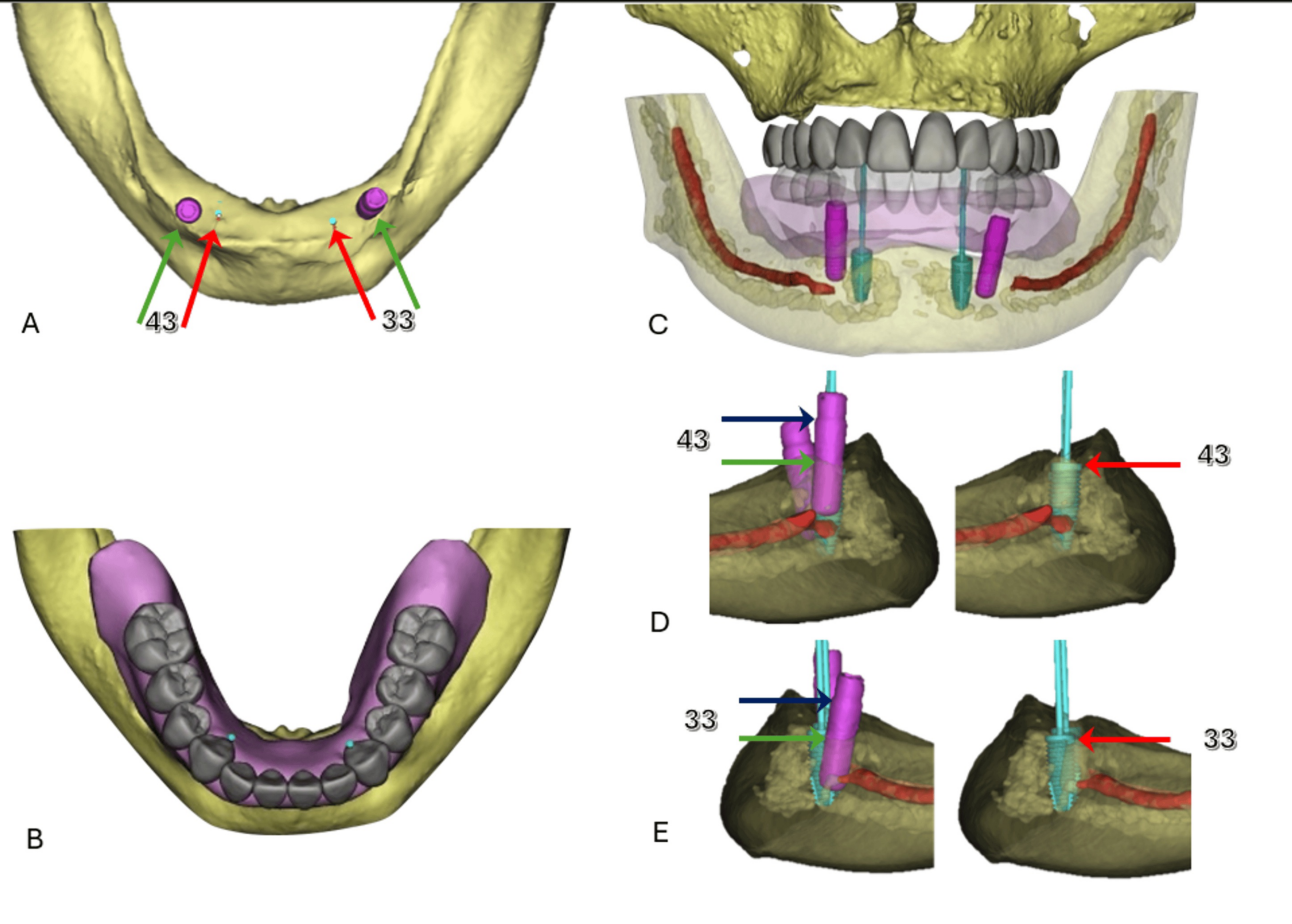
Digital planning. (A) Transverse view; (B) transverse view with simulated implant emergence; (C) frontal view with prosthetic simulation; (D-E) Sagittal views of the implants in teeth 43 and 33 showing the peri-implant bone defect with exposure of the implant threads (green arrows), the level corresponding to the platform of the previous implant (blue arrows), and the planned three-dimensional position of the new implants (red arrows). Image created with Mimics software (Materialise, Leuven, Belgium).

CBCT was performed for three-dimensional evaluation, mainly to confirm the available bone thickness and to define a prosthetically favorable position that would improve parallelism relative to the previous rehabilitation, through virtual planning with Mimics software. The transverse view (Figure 2A) was used to analyze available bone thickness and its relationship with relevant anatomical structures. The transverse view with simulated prosthetic emergence (Figure 2B) helped define the ideal axis of insertion. The frontal view with prosthetic simulation (Figure 2C) was used to verify parallelism and load distribution. Comparative sagittal views of implants 43 (Figure 2D) and 33 (Figure 2E) showed the preexisting circumferential bone defect and the planned position after explantation, supporting the placement of two new intraforaminal implants of 4.0 × 10 mm under a prosthetically guided protocol.

Surgical phase I: explantation and guided placement

**Figure 3:**
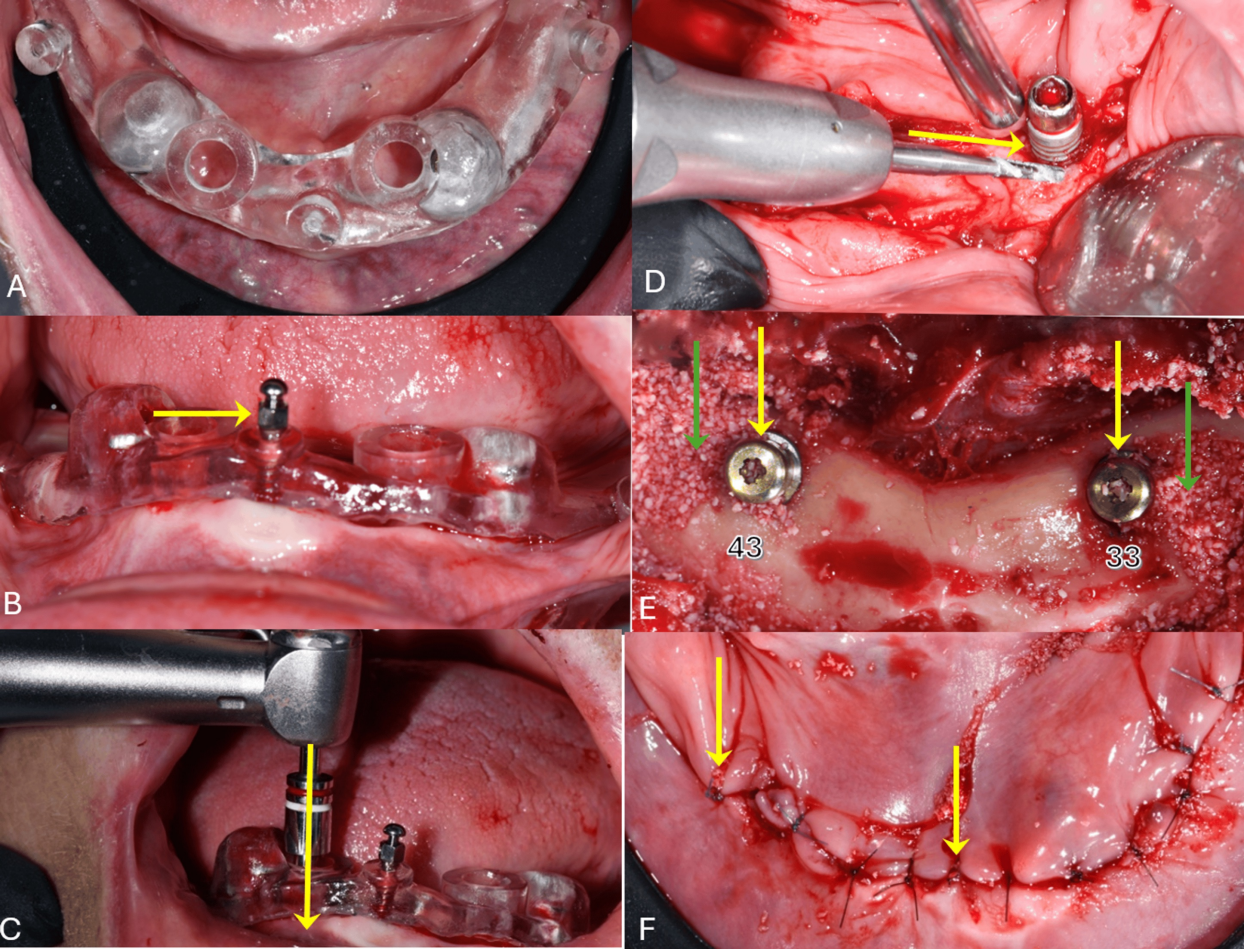
Surgical phase I. (A) Passive adaptation of the surgical guide. (B) Securing the guide with a stabilization pin (yellow arrow). (C) Guided drilling showing the preparation axis (yellow arrow). (D) Implant removal using a low-speed drill (yellow arrow). (E) Implant placement combined with guided bone regeneration using autograft and xenograft (yellow arrows: implants; green arrows: graft material). (F) Horizontal mattress suture (yellow arrows).

Local infiltrative anesthesia was complemented with bilateral inferior alveolar nerve block using 2% lidocaine with 1:100,000 epinephrine and 4% articaine with 1:100,000 epinephrine. The passive fit of the surgical guide was first verified (Figure 3A) and the guide was then secured with anchoring screws (Figure 3B). Osteotomy was performed by sequential drilling under abundant saline irrigation (Figure 3C). Atraumatic explantation of the compromised implants was performed with a low-speed reverse-torque device (Figure 3D). Two Neodent GM implants of 4.0 × 10 mm (Neodent, Curitiba, Brazil) were then immediately placed using a surgical motor with the torque limit programmed at 50 Ncm; final seating was completed with a manual ratchet up to a final insertion torque of 20 Ncm. The residual peri-implant bone defect was grafted with a combination of autologous bone particles and a xenograft (NUKBONE, Biocriss, Mexico City, Mexico) (Figure 3E). Surgical closure was performed with a horizontal mattress suture using 4-0 resorbable polyglycolic acid (Figure 3F).

Postoperative medication included amoxicillin/clavulanic acid 875/125 mg every 12 hours for seven days and ibuprofen 600 mg every eight hours for three days, considering the combined surgical procedure and the patient’s systemic conditions. Sutures were removed 15 days after surgery, and uneventful soft tissue healing was observed.

Surgical phase II: peri-implant soft tissue augmentation

**Figure 4:**
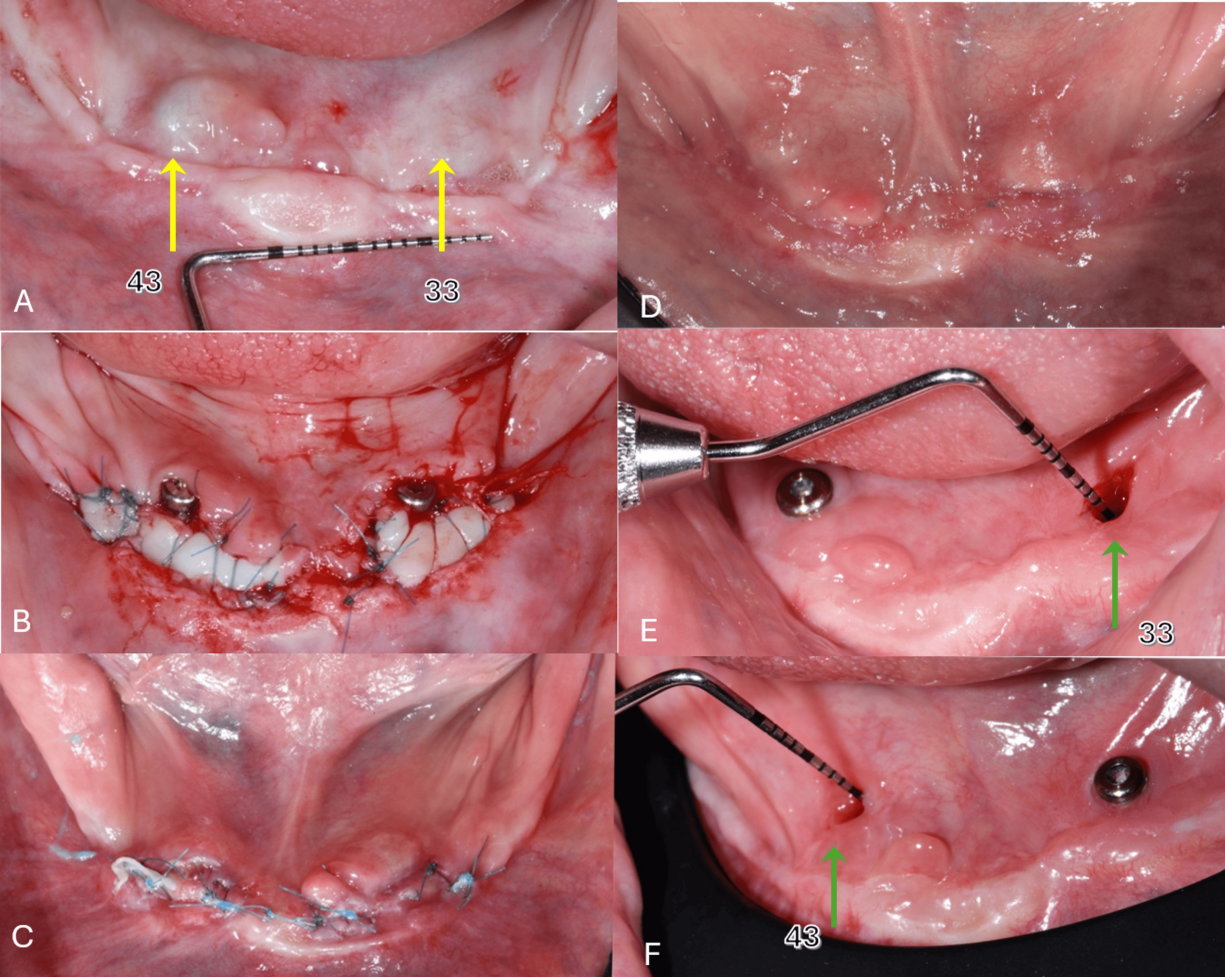
Surgical phase II. (A) Initial evaluation showing a deficiency of peri-implant keratinized mucosa (yellow arrows). (B) Placement of de-epithelialized free gingival grafts. (C-D) Postoperative follow-ups at 15 and 21 days. (E-F) Clinical evaluation showing increased thickness of the peri-implant soft tissues (green arrows).

Reassessment of the peri-implant keratinized mucosa (Figure 4A) confirmed the lack of an adequate band of keratinized tissue. De-epithelialized free gingival grafts were harvested from the palate (approximate dimensions: 18 mm in length × 8 mm in width × 1.5 mm in thickness) and secured to the recipient bed with crossed 5-0 nylon sutures, apically displacing the mucogingival line in order to gain keratinized mucosa around both implants (Figure 4B). At 15 days, favorable initial integration of the graft was observed (Figure 4C), and at 21 days the graft showed clinical stability (Figure 4D). Subsequent follow-up visits (Figures 4E, 4F) confirmed satisfactory peri-implant soft tissue augmentation, with clinically favorable tissue contour, increased soft tissue volume, and improved peri-implant tissue stability around implants 33 and 43.

**Figure 5:**
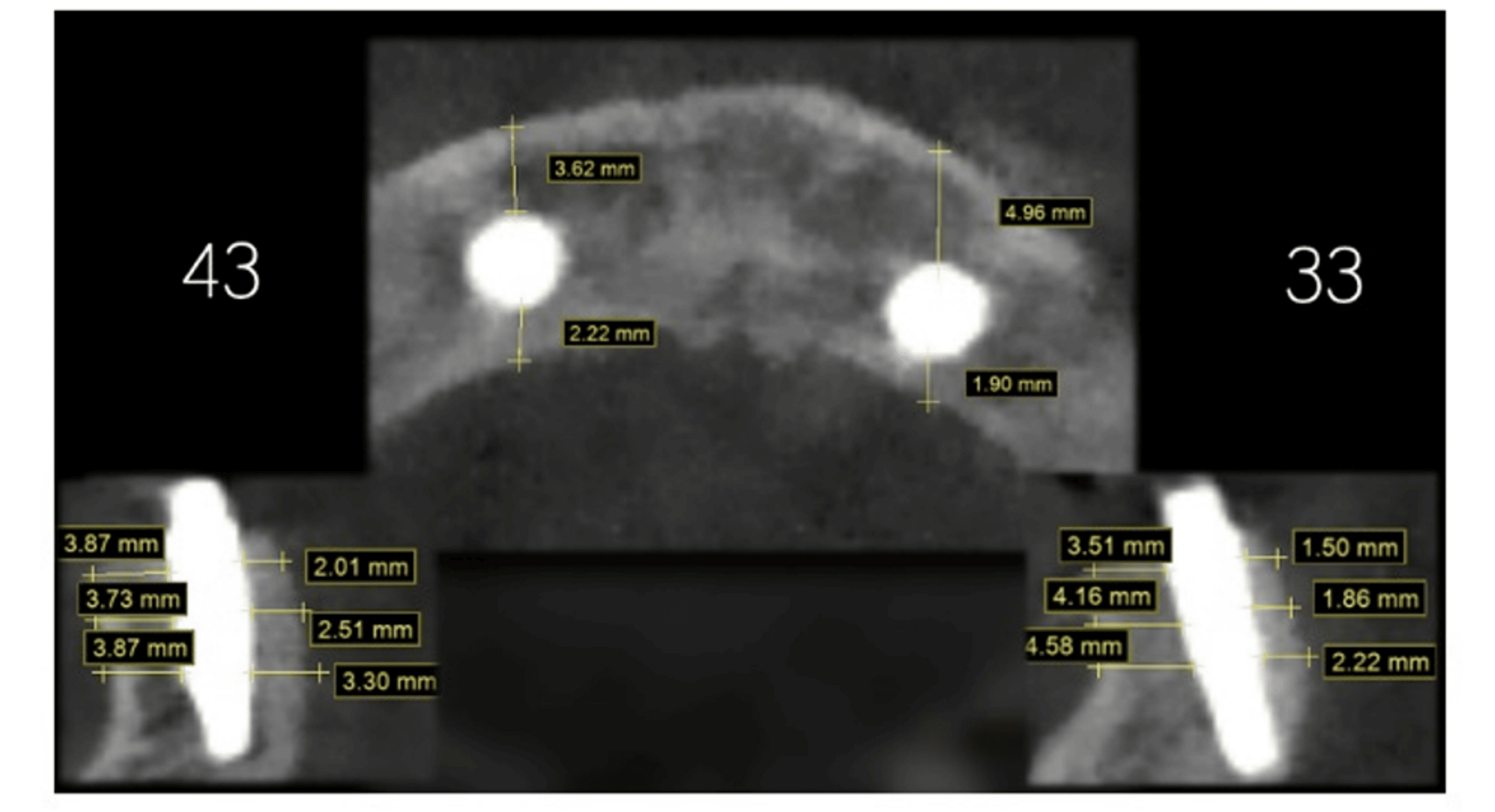
Postoperative cone-beam computed tomography confirming the three-dimensional position of the new intraforaminal implants and the absence of peri-implant radiolucency.

The postoperative CBCT confirmed an adequate three-dimensional position of the implants and the absence of peri-implant radiolucencies (Figure 5).

Prosthetic phase

**Figure 6:**
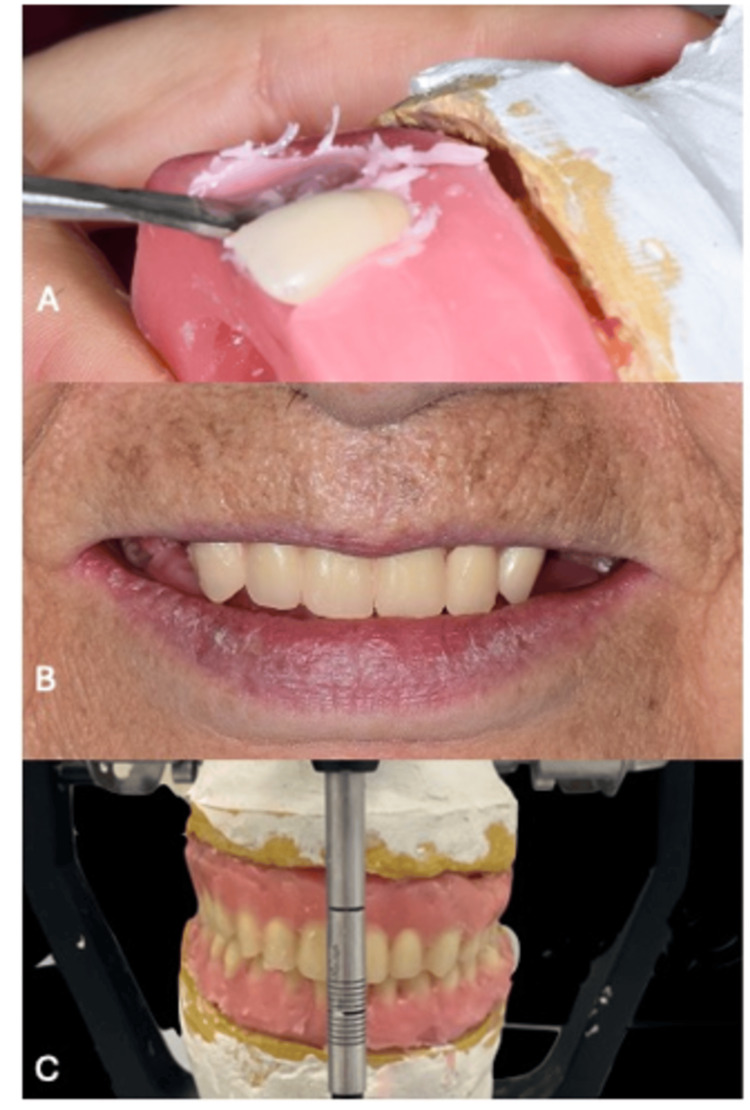
Prosthetic try-in. (A) Anterior tooth setup; (B) clinical assessment of esthetic and phonetic parameters; (C) complete tooth setup mounted on a semi-adjustable articulator.

The prosthetic phase began with anterior teeth try-in (Figure 6A) and a clinical assessment of esthetic and phonetic parameters (Figure 6B). The complete tooth setup was then mounted on a semi-adjustable articulator (Figure 6C) to verify vertical dimension and intermaxillary relationships.

**Figure 7:**
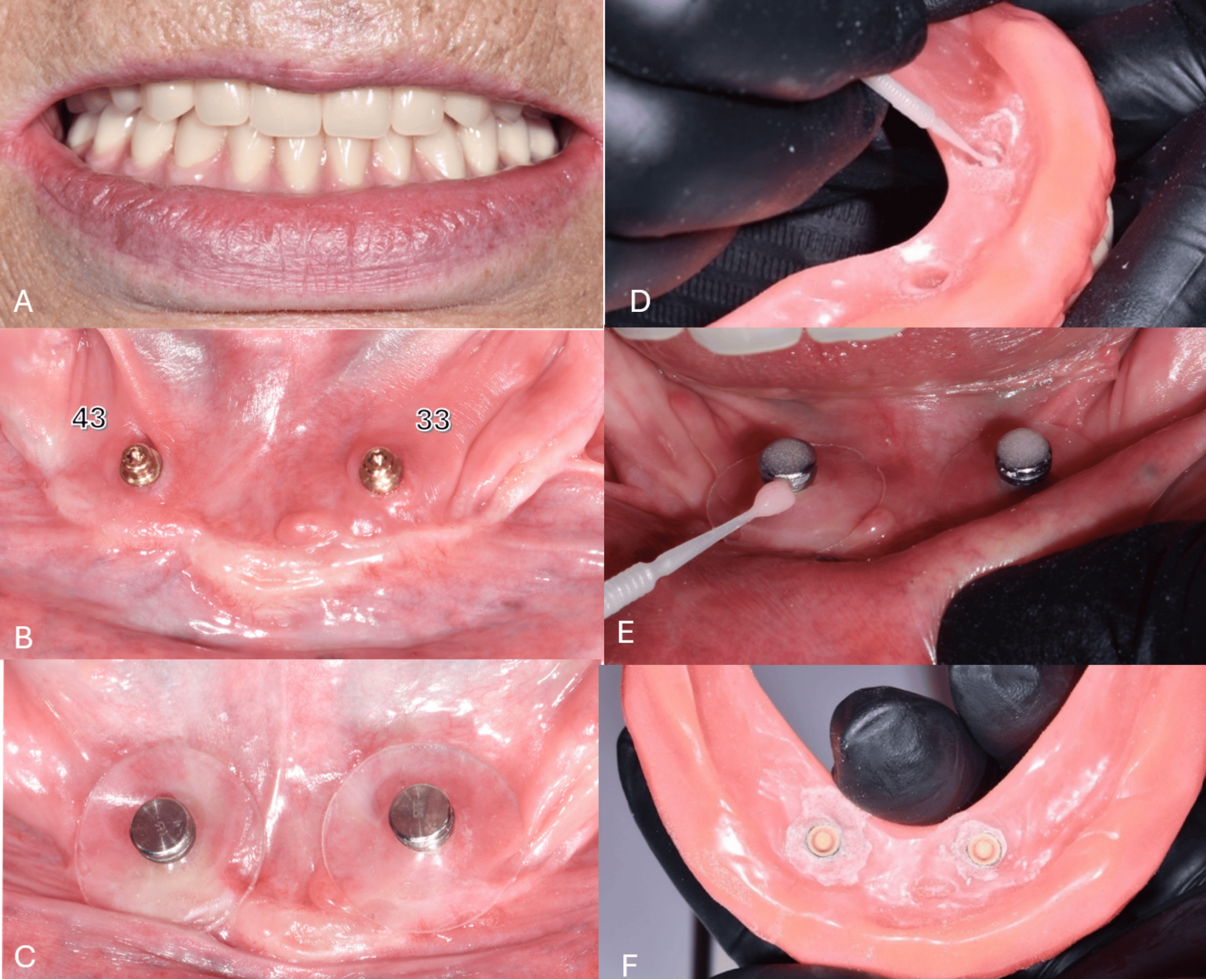
Prosthesis delivery. (A) Initial prosthesis try-in; (B) placement of Equator attachments (Rhein83, Bologna, Italy); (C) placement of protective discs; (D, E) direct chairside relining and capture of the housings; (F) final aspect of the housings within the overdenture.

At delivery, an initial prosthesis try-in was performed (Figure 7A), followed by placement of Equator low-profile attachments (Figure 7B). Protective discs were placed (Figure 7C) and the housings were captured by direct chairside relining of the overdenture intaglio surface with self-curing acrylic resin (Figures 7D, 7E). The final aspect of the housings within the overdenture is shown in Figure 7F. Light retention inserts were selected to facilitate prosthesis manipulation by the patient and to reduce loading on the recently placed implants.

Final evaluation and maintenance

**Figure 8:**
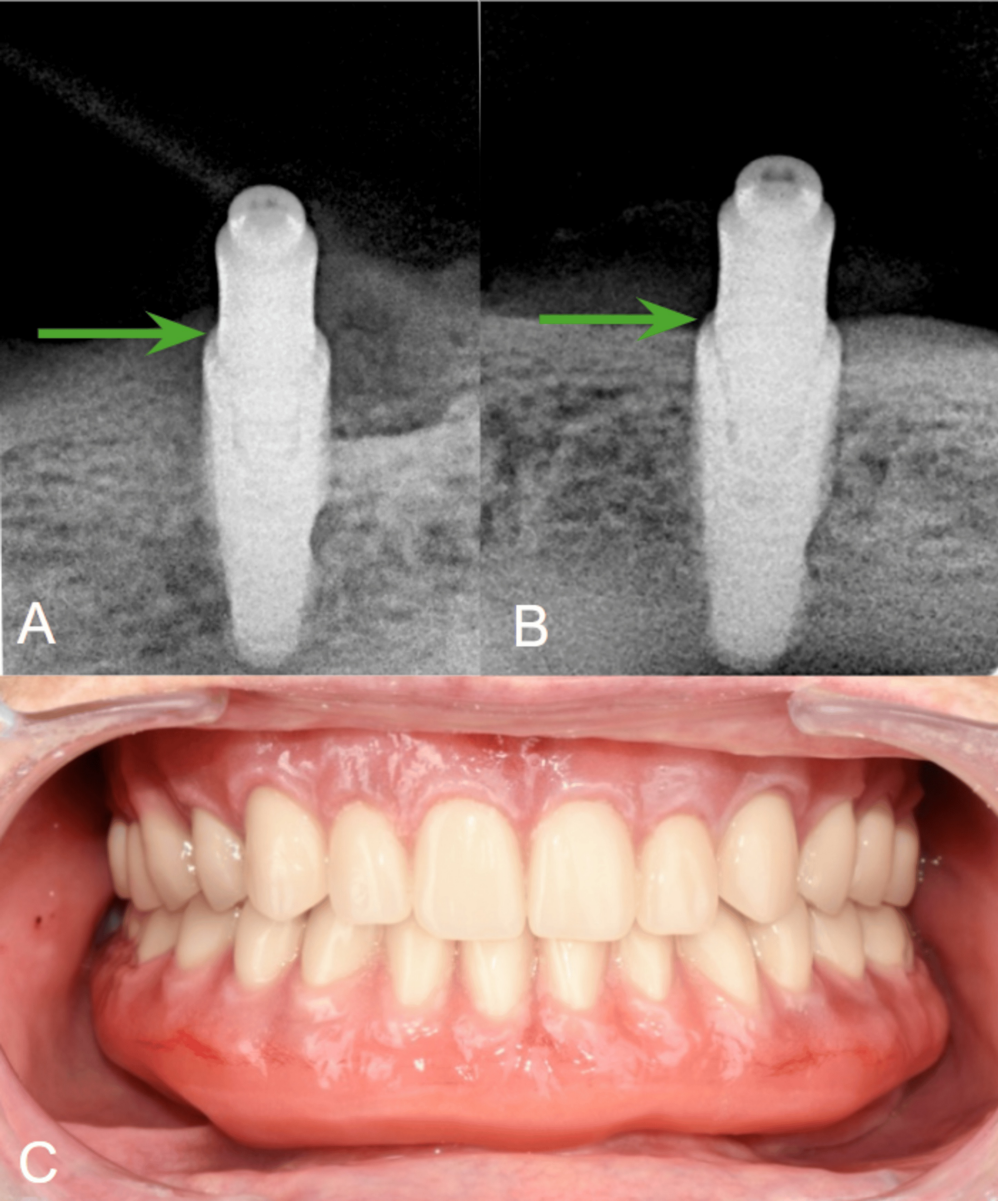
Three-month follow-up after definitive loading. (A) Periapical radiograph of implant 33; (B) periapical radiograph of implant 43 (green arrows); (C) clinical evaluation of the overdenture during the first peri-implant maintenance visit.

Radiographic evaluation three months after definitive loading showed peri-implant bone stability around implant 33 (Figure 8A) and implant 43 (Figure 8B). Peri-implant probing depths at the three-month follow-up were 3 mm at implant 33 and 2 mm at implant 43, with absence of bleeding on probing at both sites. Clinical evaluation of the overdenture during the first peri-implant maintenance visit (Figure 8C). The patient reported satisfactory function, comfort, and ease of hygiene at the last evaluation. To favor long-term peri-implant tissue stability, a structured maintenance protocol with quarterly recall visits was established, focused on clinical and radiographic monitoring and on professional biofilm control.

The overall treatment chronology, from explantation to the three-month follow-up, is summarized in Tables 1, 2. No additional vertical bone loss was observed on CBCT at the three-month follow-up for implants 33 and 43. 

**Table 1:** Chronology of the treatment phases and follow-up.

Stage / procedure	Time point / interval
Diagnostic assessment (baseline)	Day 167
Surgical phase I: explantation, guided placement, and bone grafting	Day 203
Suture removal	15 days after surgery
Surgical phase II: peri-implant soft-tissue augmentation (free gingival grafts)	[18 weeks after phase I]
Graft controls	15 and 21 days after grafting
Prosthetic delivery / definitive loading (overdenture)	[at 11 months following the initial surgical phase]
Final evaluation / first maintenance visit	Three months after definitive loading

**Table 2:** Baseline and three-month clinical and radiographic findings Baseline versus three-month peri-implant clinical and radiographic findings (implants 33 and 43). Final insertion torque 20 Ncm for both implants (4.0 × 10 mm Neodent GM (Curitiba, Brazil); torque limiter 50 Ncm).

Parameter	Baseline (33 / 43)	Three months (33 / 43)
Peri-implant probing depth (mm)	7 / 5	3 / 2
Bleeding on probing	Present / Present	Absent / Absent

## Discussion

Peri-implantitis is one of the most relevant biological complications of implant therapy, with reported prevalence rates of approximately 22%, while peri-implant mucositis may affect up to 43% of cases [7]. In the present case, advanced circumferential bone loss together with the absence of an adequate band of keratinized mucosa was consistent with risk factors that have been described in the literature as being associated with the development and progression of peri-implant disease [12,15].

The defect classification proposed by Monje et al. [12] integrates morphological and severity components based on three-dimensional radiographic assessment and is particularly useful in clinical decision-making for advanced peri-implantitis. According to that system, combined defects (Class III) include both an intraosseous and a supracrestal component, and the “c” subclass corresponds to a circumferential pattern. In the present case, the combination of an advanced Class IIIc defect at implant 33 with extensive vertical bone loss exposing the implant threads provided objective grounds to consider the prognosis of that implant as unfavorable, in line with previous reports describing limited predictability of regenerative therapy in this defect configuration.

The long-term success of dental implants is not determined exclusively by initial osseointegration and depends largely on structured maintenance programs. Periodic clinical evaluation, radiographic monitoring, and professional biofilm control are essential to preserve peri-implant health [5]. In the present patient, the absence of regular professional follow-up may have contributed to the silent progression of the inflammatory process, a pattern previously reported in patients rehabilitated with mandibular overdentures retained by intraforaminal implants [11]. 

The systemic profile of this patient warrants particular emphasis, as it is central to the complexity of the case. Primary Sjögren’s syndrome produces salivary gland dysfunction and hyposalivation; the resulting reduction in salivary flow compromises mechanical biofilm clearance and the antimicrobial and buffering functions of saliva, fostering an oral environment that favors microbial colonization around teeth and implants. Rheumatoid arthritis, a chronic immune-mediated disease frequently managed with immunomodulatory agents, may further alter the host inflammatory response and impair wound healing. In a patient already presenting advanced peri-implant bone loss and a deficient band of keratinized mucosa, these factors were judged to reduce the predictability of conservative or regenerative approaches and reinforced the decision to explant the compromised implants and to integrate soft-tissue augmentation and a structured maintenance protocol into the treatment plan.

When peri-implantitis progresses to advanced stages with extensive bone defects, the prognosis of the implant becomes uncertain [8]. In the present case, advanced circumferential bone loss, exposure of the implant threads, and the patient’s systemic conditions supported a limited prognosis for regenerative therapies, and explantation was therefore considered the most predictable therapeutic alternative. Although regenerative therapies have been proposed for the treatment of peri-implantitis, their success depends largely on defect morphology and on adequate control of risk factors. Zhou et al. reported that placement of new implants at sites of previous failure is clinically feasible and may be associated with favorable survival rates when proper decontamination and site planning are performed [16]. As a complementary observation, Covani et al. reported that atraumatic removal of compromised implants helps preserve residual bone volume and optimize site conditions for subsequent rehabilitation [17]. In addition, immediate placement reduced the number of surgical interventions and made it possible to optimize the three-dimensional position of the implants in a medically compromised patient.

Prosthetically guided immediate placement allowed the three-dimensional position of the new implants to be optimized, parallelism to be improved, and the total treatment time to be reduced, in line with previous clinical reports. Implant placement at sites previously affected by infection nevertheless remains a debated topic in the literature [16]. The approach used here is based on the concept of prosthetically driven implant dentistry, in which planning is guided by the final prosthetic requirements, with the aim of optimizing the three-dimensional position of the implants and improving the predictability of functional and biomechanical outcomes. Compared with the previous rehabilitation, this planning made it possible to correct prosthetic emergence and implant alignment, factors that may have contributed to the initial failure.

According to the McGill Consensus, an implant-retained mandibular overdenture supported by at least two implants is considered the minimum standard of care for patients with mandibular edentulism [9]. Compared with conventional complete dentures, this treatment modality has shown greater prosthetic stability, higher patient satisfaction [10], and high long-term implant survival rates [18]. Implant-retained overdentures, however, also present specific challenges related to peri-implant maintenance and biofilm accumulation in retention components [10,11]. Studies evaluating the retentive characteristics of different attachment systems used in mandibular overdentures have reported adequate retention and favorable mechanical behavior in axial low-profile systems [19]; nevertheless, these components require careful hygiene and periodic follow-up to limit the recurrence of peri-implant inflammation [11]. The Equator system was selected because of its low vertical profile, which is advantageous in cases with limited interarch space, and because of the relative ease of insertion and removal of the prosthesis, characteristics that may help an elderly patient manage the prosthesis independently. Light retention inserts were chosen to facilitate prosthesis manipulation.

The decision to place two intraforaminal implants, rather than a greater number of implants with a bar or a fixed full-arch prosthesis, was based on the McGill consensus [9] and on patient-specific factors, including the atrophic mandibular ridge (Atwood Class IV), the available intraforaminal bone, the patient’s age and manual dexterity, the need for a hygiene-friendly and easily handled prosthesis, and cost-effectiveness. A conventional complete denture was considered insufficient given the reported instability and functional impairment, whereas more extensive fixed rehabilitation was deemed less favorable in view of the systemic conditions and the associated maintenance demands.

It should be emphasized that, in a two-implant overdenture retained by resilient axial attachments, the implants chiefly provide retention and lateral stabilization, while functional masticatory loads are shared with the posterior residual ridge and its overlying mucosa. This load-sharing principle, reflected in the implant-mucosa-supported designation adopted here, guided both the selection of low-profile resilient attachments and the use of light retention inserts to limit loading on the recently placed implants.

The management of peri-implant soft tissues was another relevant aspect of this case. Although the minimum required width of peri-implant keratinized mucosa remains under discussion [15,20], several studies suggest that an adequate band of keratinized tissue is associated with less peri-implant inflammation and better biofilm control. In a systematic review with meta-analysis, Ravidà et al. reported that implants with a narrow band of keratinized mucosa showed greater biofilm accumulation and a higher probability of peri-implant inflammation [15]. Free gingival grafting in the present case was aimed at improving patient comfort during hygiene and at promoting a more stable peri-implant environment, in line with the available evidence [15,20].

The integrated approach combining elimination of the infectious focus, prosthetically guided planning, peri-implant tissue management, and structured maintenance allowed favorable clinical conditions to be re-established [5,8]. As a strength, this case illustrates the feasibility of an implant-mucosa-supported rehabilitation through immediate placement at a previously compromised site, integrating surgical and prosthetic decisions within a single therapeutic plan. Several limitations should also be acknowledged. This is a single clinical case with a follow-up of only three months after definitive loading, which is insufficient to draw conclusions regarding long-term peri-implant stability or implant survival. The findings are specific to this patient and should not be extrapolated to broader populations or used to define general treatment protocols. In addition, no quantitative volumetric assessment of peri-implant bone changes was performed, no microbiological analysis was conducted, and patient-reported outcomes were collected informally rather than through validated instruments such as the Oral Health Impact Profile for Edentulous Patients (OHIP-EDENT) [21]. Clinical studies with larger samples and longer follow-up are needed to confirm the predictability of explantation followed by prosthetically guided immediate placement and soft tissue augmentation in patients with advanced peri-implantitis.

## Conclusions

Implant-retained mandibular overdentures supported by two intraforaminal implants remain a predictable rehabilitation option for patients with mandibular edentulism when prosthetically guided planning, adequate management of peri-implant tissues, and a structured maintenance protocol are integrated into the treatment plan.

In the patient described here, explantation of two implants compromised by an advanced Class IIIc combined peri-implant defect, followed by immediate placement of new implants and soft tissue augmentation, was associated with re-establishment of favorable peri-implant conditions and recovery of prosthetic stability and function.

Although the short-term outcomes were satisfactory, long-term stability will depend on biofilm control and on periodic clinical and radiographic follow-up. The findings should be interpreted with caution given the single-case design and the limited follow-up; even so, they support the role of prosthetically guided planning as a relevant component of peri-implant rehabilitation in medically compromised patients.

Accordingly, these findings should not be generalized; longer follow-up and larger case series are needed to confirm the predictability of explantation followed by prosthetically guided immediate placement and soft-tissue augmentation.
